# Medical Geological assessment of fluoride contaminated groundwater in parts of Indo-Gangetic Alluvial plains

**DOI:** 10.1038/s41598-019-52812-3

**Published:** 2019-11-07

**Authors:** Suresh Kumar, Rambabu Singh, A. S. Venkatesh, G. Udayabhanu, P. R. Sahoo

**Affiliations:** 1 Central Ground Water Board, Patna, India; 2 Central Mine Planning and Design Institute Limited, Bilaspur, India; 3 Department of Applied Geology, Indian Institute of Technology, (Indian School of Mines), Dhanbad, India; 4 Department of Chemistry, Indian Institute of Technology (Indian School of Mines), Dhanbad, India

**Keywords:** Environmental impact, Environmental social sciences, Hydrology, Risk factors

## Abstract

As drinking water is considered as a major pathway of exposure to fluoride in the human body, an endeavor has been made for the assessment of the non-carcinogenic health risk by using hazard quotient (HQ) of fluoride for males, females, and children separately in fluoride affected ground water areas of Indo-Gangetic Alluvial Plains. The study suggests that children groups are more prone to the non-carcinogenic risk of fluoride in the area as HQ for fluoride is more than unitary in 44% (Pre-monsoon) and 38% (Post-monsoon) samples respectively. Field survey conducted in fluoride-affected villagers of the study area portrays cases of mottling of teeth and bone deformities depending on the duration and dosage of fluoride consumption. Petrographic observations of host rocks coupled with molar ratios of chemical species studies exemplify that weathered material developed over the granite-gneiss, mica-schist, amphibolite, granitic intrusive and pegmatite veins due to weathering and extensive water-rock interaction resulting higher concentration of fluoride in groundwater. Likewise, the base exchange index (r1) and meteoric genesis index (r2) advocates that most of the samples belong to Na^+^-HCO_3_^−^ type and meteoric origin respectively, and substantiate longer residence time of water along with solute acquisition processes are responsible for elevated fluoride in groundwater. It is, therefore, solar energy-driven electrolytic de-fluoridation technology ought to be provided on a priority basis to the affected inhabitants besides the implementation of rainwater harvesting schemes for mitigation/ dilution of elevated fluoride concentration.

## Introduction

Several clinical studies and researches have been conducted to find out the impact of fluoride concentration in drinking water on human health. It is a well-known fact that fluoride (F^−^) at low concentration (<0.5 mg/L) induces dental caries, which enhances the risk of tooth decay^1,2^. On the other hand, ingestion of excessive fluoride imparts adverse effect on human health^3^. It is a well-established fact that elevated F^−^ concentration in drinking water is linked to dental and skeletal fluorosis depending on the amount of intake and duration of exposure^4,5^. Excessive fluoride can also damage structure, function and metabolism of soft tissues such as kidney, liver, lung and testicles^6–9^. Various clinical studies suggest that ingestion of excessive fluoride promotes physiological dysfunctions like mutagenesis, immune suppression, carcinogenesis and growth retardation^3,10^. At times, higher concentrations of fluoride can also promote neuro-toxicological effects^11,12^. Several studies indicate that exposure of excessive fluoride does impart the vulnerable effect on the mental ability of the children. Choi *et al*.^13^ suggests that children with exposure to high fluoride were found with significantly lower IQ compared to children residing in low fluoride areas^13^. Fluoride easily reaches the placenta, and exposure to fluoride may permanently harm the foetus^14^. In the human body, the thyroid gland is the most sensitive tissue to the fluoride and exposure to fluoride raises TSH (thyroid stimulating hormone) concentration and decreases T_3_ and T_4_ concentration, thereby resulting in hypothyroidism^15,16^. In several studies, it has been pointed out that prolonged exposure of fluoride from drinking water does develop insulin resistance in human beings^17,18^. Excessive consumption of fluoride ultimately leads to hyperglycemia and impaired glucose tolerance^19,20^. While some studies showed that exposure to high fluoride may induce adverse impact on human reproductive system, leading to infertility problems and low body weight of neonates^3,21,22^. Osteosarcoma is known as a potential cancer target site due to fluoride deposition in bone^23,24^. Cohn (1992) observed that the association of fluoride in public water with an increased incidence of osteosarcoma in young males^25^.

Ingestion of fluoride to human body, mainly occurs via, water and through other food and hygiene related foodstuffs, drugs, toothpaste. Among these, drinking water is the most prominent source of human contact with fluoride^26^. About 90% of the ingested fluoride in drinking water is absorbed, particularly from the gastrointestinal tract when the fluoride concentration increases by 1 ppm^27^. On the contrary, only 30–40% of fluoride from food sources gets absorbed in the digestive system^28^. Hence, it is essential to identify the places of excessive fluoride concentration in groundwater and to assess health risk of exposure to fluoride. Health risk is an important assessment method applied to examine the real risk on human health resulting from consumption of specific chemical agents during specified time period^29^. The incidence of excessive fluoride in groundwater is intense and alarming in the Indian states of Telangana, Bihar, Gujarat, Madhya Pradesh, Rajasthan, Tamil Nadu, Assam and Uttar Pradesh^30^. In the present study area which is a part of Jamui district of Bihar state in India, where excessive fluoride concentration has been reported in groundwater in parts of Indo-Gangetic Alluvial Plains where population density is relatively high^31^.

The principal objective of this study is to (1) outline the geochemical mechanisms responsible for the fluoride enhancement in the aquifers of study area vis-à-vis plausible cause(s) and source characterization with the aid of field geological and petrographic parameterization of host rocks, (2) assessment of health risk from fluoride ingestion through drinking water for males, females and children (3) displaying GIS-based spatial and temporal distribution of fluoride and identification of risk areas for taking preventive measures. Therefore, the outcome of the medical geological study would be helpful in chalking out future and long-term planning for augmenting safe drinking water resources, especially in areas, where groundwater is stressed and affected by fluoride contamination with a view to consequently minimize the adverse health impacts due to excessive fluoride exposure.

## Geological and Hydrogeological Assessment of Study Areas

The study area is located between North latitude 24°40′ to 25^0^10′ and East longitude 85^0^50′ to 86^0^35′ is a part of Jamui district in Bihar state, covers large tract of Indo-Gangetic Alluvial Plains. It covers about 1791 km^2^, with six administrative blocks: Laxhmipur, Sikandra, Khaira, Gidhour, Sono, Barhat and Jhajha. The hot weather starts in March and continues till middle of June. Generally rains commence in June and lasts till October. A tropical monsoon climate prevails in the area with an average annual rainfall of study area is nearly 1042 mm and most of the rainfall is received during June to September due to south-west monsoon. Catchments of Kiul River and Barnar River form a major part of the study area and the Sukhnar, Bernar and Ulai streams drain this region. The Nagi stream flows to the west and finally joins the Ulai River near Patsanda and all the streams named above, become dry during summer.

**Figure 1:**
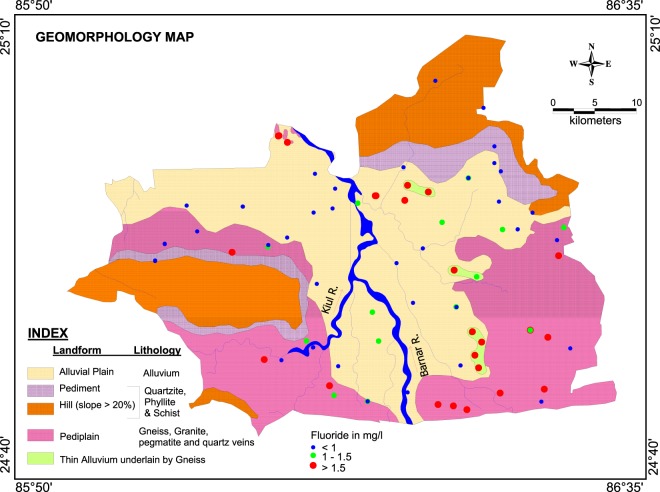
Map illustrating location of groundwater samples alongside geology and geomorphology of the study area.

The area comprises of diverse geomorphology and major geomorphologic units are rocky upland, pediplain and alluvial plain (Fig. 1). Maximum height of 475 m above mean sea level has been found in Barhat block. The rolling topography of pediplain consists of relief up to 300 m above mean sea level. Alluvial fills in the study area is a part of Jamui Formation which constitutes the oldest continental quaternary deposits and is generally termed as “older alluvium” in Indian geology^32^. The thickness of the alluvium increases towards north along the river courses finally merging with the Gangetic alluvium, south of the river Ganga. Thickness of the alluvium varies from 90 m in northern part to 10 m in southern part^33^. The major rock types found in this region are quartzite, quartz-mica schist, biotite-muscovite schist, hornblende schist, granite, composite gneisses, pegmatites, amphibolite and quartz veins. Exposure of biotite/muscovite-schists occurs in the narrow zone between the Barnar and Ulai streams. Massive granitic bodies are seen mostly as inselbergs. Medium grained granites are exposed as small bands/layers from 1.5 km west of Tarakura to 1.2 km SE of Korwadih. Biotite gneisses form a narrow zone from west of Bhitra to west of Lalmatia and is also seen south-east of Bhelbinda, Karhara and east of Dhamna. Medium to coarse-grained, grey to pink and composite biotite gneisses have also been encountered at some places.

The most potential aquifer in the study area is found in the valley-fill material. It consists of fine, medium as well as coarse sands. Due to humid climate, chemical weathering is also prevalent in the area. As a result, moderately thick weathered mantle has been formed due to weathering of crystalline rocks. Depth to water level is somewhat higher in the weathered zone than alluvium zone. Highly fractured crystalline rocks are also good depository/repository of groundwater in the area. CGWB drilled wells of this area confirms three to four sets of fractures/ joints occurring at different depths up to 200 m bgl (below ground level).

## Materials and Methods

### Sample collection metrics and analytical approaches

Seventy six groundwater samples were collected in May 2014 and 53 groundwater samples were collected in November 2014 from shallow and deep wells respectively, for estimation of major ions. Ninety samples were collected in both seasons (2014) for estimation of fluoride separately. Out of these, 22 samples belonged to shallow aquifer and remaining were collected from deeper aquifer. Also, resampled again in 33 random sites during May, 2018 and there is no major change in the obtained results (Supplementary Fig. S1). Groundwater samples were collected in high density polyethylene bottle (HDPE) which were rinsed twice with distilled water before collection of water samples. All the chemical parameters were estimated within two weeks after collection of samples using standard procedures as given by American Public Health Association^34^.

Sodium and Potassium were estimated in the groundwater samples using flame photometer (Systronics 128). Fluoride in the water samples were analysed following SPADNS [4,5-Dihydroxy-3-(p-sulfophenylazo)-2,7-naphthalene disulfonic acid, trisodium salt] colorimetric method by using double beam spectrophotometer (Systronics 2202). Analysis of water samples for bicarbonate and carbonate was carried by using titrimetry method. Total hardness (TH) as CaCO_3_, Calcium and Magnesium were analysed using EDTA method. All chemical parameters, except pH (unit) and EC (µs/cm), have been expressed in terms of mg/L. The analytical precision of data was ensured by screening water samples having ionic charge balance beyond ±5%. The Quality control was achieved by using duplicate sub-samples and standard materials.

### Fluoride hazard quotient and medical geological health implications in divergent human populace

Medical geology of the area, combining health risk assessment is an effective method to estimate the probability and content of adverse impact on human health due to exposure of a specific chemical for a specific duration^29,35–37^. Selinus *et al*.(2018)^38^ advocated risk assessment of human health on exposure of any chemical comprises to be of four stages. These are hazard identification, toxicity reference values selection, exposure assessment, and risk characterization. In this study, assessment of human health risk is undertaken based on exposure to fluoride and overall medical geological environment has been outlined. There are many ways of exposure to fluoride, for instance, consumption of drinking water, intake of other beverages and food, oral soil intake, inhalation of dust and ingestion of fluoride rich toothpaste, though of late this practice is discontinued^11^. It has been emphasized in several studies that drinking water is the prominent source of exposure to non-carcinogenic risk by fluoride^39^. Therefore, health risk associated with daily consumption of excess fluoride water has been calculated for the present study area by using following formula (1)^40^:

1 $${\rm{Chronic}}\,{\rm{daily}}\,{\rm{intake}}\,({\rm{CDI}})=[{\rm{Cw}}\ast {\rm{IR}}\ast {\rm{EF}}\ast {\rm{ED}}]/[{\rm{BW}}\ast {\rm{AT}}]$$

where CDI is chronic daily intake of water consumed per day by drinking water (mg/kg/day), C_w_ is the concentration of fluoride in drinking water (mg/L), IR is the drinking water ingestion rate (L/day), EF is the exposure frequency (day/year) and ED is exposure period (years), BW is average body weight (kg) and AT denotes average exposure time (days). (The standard reference values of all these components used in the assessment is furnished in Supplementary Table S1).

The non-carcinogenic risk to human health caused by exposure to fluoride is expressed in terms of hazard quotient (HQ) and is calculated using formulae (2):

2 $${\rm{HQFluoride}}=\frac{{\rm{CDI}}}{{\rm{RfD}}}$$

In the above formulae RfD denotes the reference fluoride dose in mg/kg/day. RfD is used to assess the health risk of fluoride during a specific exposure pathway. According to Integrated Risk Information System (IRIS) of USEPA, the amount of Rfd through oral ingestion and drinking water is 0.06 mg/kg/day (0.05 water and 0.01 diet)^6^. HQ value less than one ensures safety from the risk of adverse health implications, whereas, if HQ value exceeds unitary value, there may be potential non-carcinogenic health effects due to ingestion of fluoride contaminated water.

## Results and Discussion

### Role of major ion chemistry and implications on health

The statistical parameters like minimum, maximum and % of groundwater samples below permissible limit as per BIS,2012^41^ for various chemical constituents in pre and post-monsoon are presented in Supplementary Table S2.There is a noticeable difference between chemical constituent of shallow and deeper aquifer. In pre-monsoon, groundwater exceeds the maximum permissible limit of sodium concentration (200 mg/L)^41^, in 44% shallow wells and 17% deeper wells, respectively, while in post-monsoon, 13% shallow and 17% deeper wells exceed this limit of 200 mg/L. In pre-monsoon 36% and in post-monsoon 30% groundwater samples exceeds magnesium concentration compared to calcium. Once calcium reaches super-saturation, it tends to precipitate resulting in excess of magnesium in the solution in comparison to calcium. The means of NO_3_,Cl and SO_4_ in shallow aquifers in pre-monsoon are 28.13 mg/L,103.5 mg/L and 55.81 mg/L respectively whereas, in deep aquifers the respective values are 22.3 mg/L,103.46 mg/L and 35.06 mg/L. About 13% groundwater samples in pre-monsoon and 18% in post-monsoon exceed the required acceptable limit of 250 mg/L chloride^41^. Higher chloride values have been noticed in shallow aquifer water samples in comparison to deeper groundwater samples. High evaporation and local contaminants may have been contributing to elevated chloride concentration in shallow aquifers^42^.

Anthropogenic sources of pollution may be the reason for higher nitrate concentration in the shallow aquifer groundwater samples as there is no evidence of geogenic origin of nitrate in the study region^43^. In pre-monsoon, fluoride concentration ranges from 0.01 mg/L to 4.8 mg/L with a mean of 1.06 mg/L in shallow aquifers and from 0.01 mg/L to 5.8 mg/L with a mean of 1.38 mg/L in deep aquifers. While in post-monsoon, fluoride concentration ranges from 0.02 mg/L to 5.54 mg/L with a mean of 0.98 mg/L in shallow aquifers and from 0.01 mg/L to 5.65 mg/L with a mean of 1.23 mg/L in deeper aquifers. A total of 19% and 18% groundwater samples collected from shallow aquifers exceed the permissible limit of 1.5 mg/L^41^ in pre and post-monsoon respectively. In deep aquifer, 34% and 28% groundwater samples are beyond the permissible limit of 1.5 mg/L before and after precipitation, respectively. Therefore, consumption of groundwater from respective sites where Na, K, NO_3_ and F exceeded the permissible limits may cause hypertensive effects, bitter taste, methaemoglobinaemia, skeletal and dental fluorosis respectively.

### Classification and genesis of groundwater vis-a-vis contamination

The prevailing dominant groundwater facies in the study area has been identified based on the content (meq/l) of selective dominant anions (Cl^−^ and SO_4_^2−^) and a cation (Na^+^) after referring to the Soltan^44^ base- exchange indices. Here the groundwater classification has been made using the following Eq. 3, where all units are in meq/L.

3 $${{\rm{r}}}_{1}={{\rm{Na}}}^{+}-{{\rm{Cl}}}^{-}/{{\rm{SO}}}_{4}^{2-}$$

**Figure 2:**
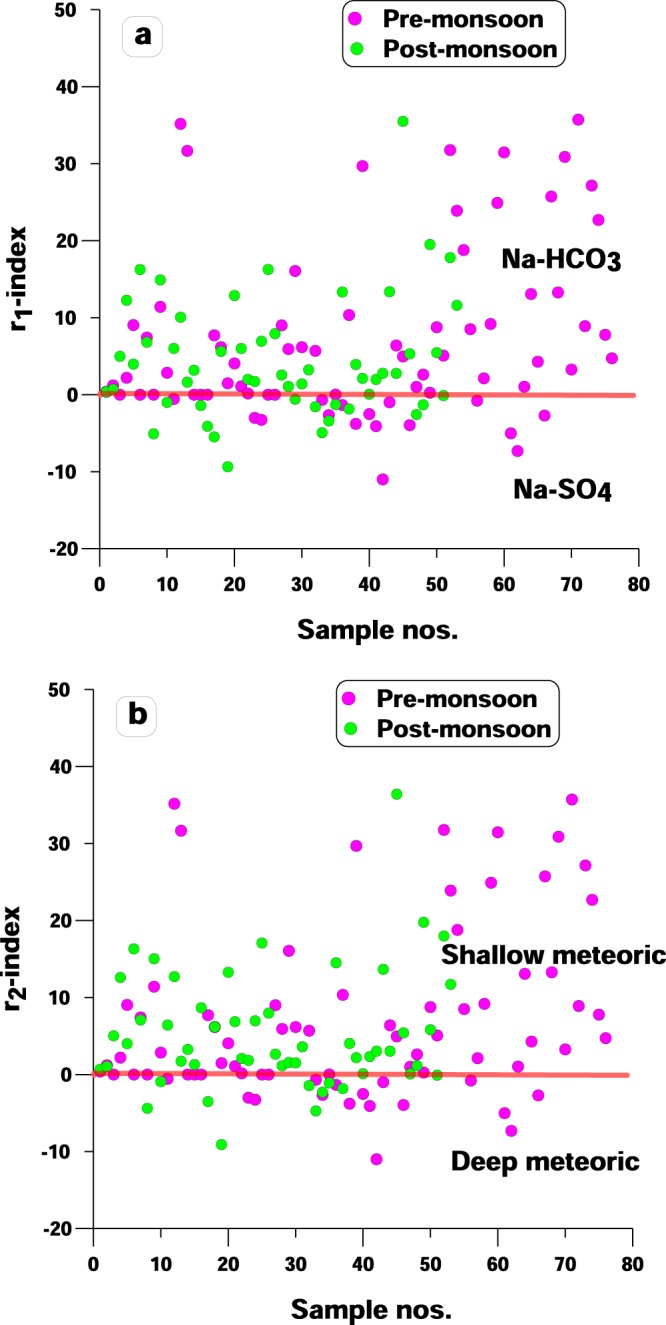
(**a**) Base exchange index (r_1_) plot (**b**) Meteoric genesis index (r_2_) plot.

In the above mentioned equation, r_1_ value more than unitary (>1) signifies groundwater is Na^+^-HCO_3_^−^ type, on the contrary, r_1_ value less than unitary (<1) denotes that groundwater is representing Na^+^-SO_4_^2−^ type. It is clear from the base exchange index (r_1_) plot (Fig. 2a) that most of the samples belong to Na^+^-HCO_3_^−^ type while 28% sample in post-monsoon and 23% sample in pre-monsoon belong to Na^+^-SO_4_^2−^ type. It is well established that the Na-HCO_3_ type water accelerates the precipitation of calcite, accompanying the release of fluoride from minerals in gneissic basement rocks and granitic intrusive due to dissolution of silicates and consequently enhancing fluoride concentration in groundwater^30,45,46^. The mineral reaction followed by ion-exchange reactions are the key governing factors responsible for elevated fluoride concentration reported in the study area^39^:

4 $${{\rm{CaF}}}_{{\rm{2}}}+N{a}_{2}C{O}_{3}\leftrightarrow \,CaC{O}_{3}+2F{}^{-}+{{\rm{2Na}}}^{+}$$

5 $${{\rm{CaF}}}_{{\rm{2}}}+2NaHC{O}_{3}\to \,CaC{O}_{3(aq)}+2N{a}^{+}+2{F}^{-}+{{\rm{H}}}_{{\rm{2}}}{\rm{O}}+{{\rm{CO}}}_{{\rm{2}}}$$

In general, dynamic groundwater resource gets replenished each year depending on hydrogeological and meteorological conditions. In the present study, meteoric genesis index (r_2_) as suggested by Soltan^44^ is used for the detection of source of groundwater (Eq. 4).

6 $${{\rm{r}}}_{2}=({{\rm{K}}}^{+}+{{\rm{Na}}}^{+})-{{\rm{Cl}}}^{-}/{{\rm{SO}}}_{4}^{2-}$$

According to this index, r_2_ value less than one (<1) indicates groundwater belongs to deeper meteoric percolation while r_2_ value more than one (>1) denotes shallow meteoric percolation. As is evident from Fig. 2b, majority of the samples in both seasons are dominated by shallow meteoric percolation (i.e. 89% groundwater samples in pre-monsoon and 87% groundwater samples in post-monsoon). It may be concluded from this that addition of fluoride in the solution is due to its prolonged retention time in the aquifer matrix and not from meteoric sources^31^. It has also been endorsed in some more studies that fluoride concentration generally banks on the extent of water- rock interaction as fluoride primarily originates from geogenic sources^5,45,47^.

### Spatio-temporal and depth-wise distribution of fluoride

**Figure 3:**
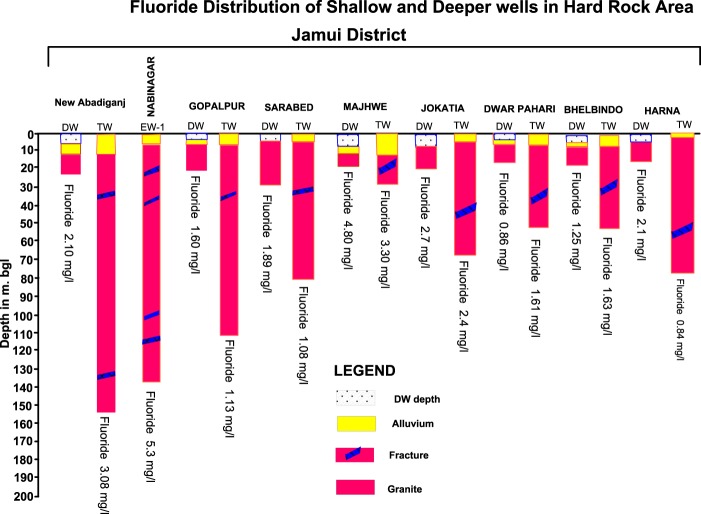
Fluoride distribution in paired deep and shallow wells of same location.

In the shallow aquifer 23% water samples and in deep aquifers 34% water samples have fluoride concentration more than the maximum permissible limit of 1.5 mg/L (Supplementary Fig. S2a and 2b). It is found that groundwater samples from hard rock areas are more enriched in fluoride concentration than groundwater samples from alluvial areas. For comparing fluoride concentration in deeper and shallow wells, some groundwater samples were collected from paired deep and shallow aquifers of same location. It is observed that in paired samples of hard rock areas, both shallow as well as deeper aquifers are enriched with fluoride in solution (Fig. 3). It has been noticed that areas with high concentration of fluoride in deeper wells are also paired with high concentration of fluoride in shallow wells. Occurrence of high fluoride levels in shallow wells may be attributed to weathering of fluoride bearing material from granite, granite-gneiss, amphibolite and mica-schist and deposition of same over the host rock as a weathered mantle might be the prominent reason for higher fluoride concentration in the area^39,47^. Upward movement of fluoride-concentrated water from deep aquifer may be another dominant factor for enrichment of fluoride concentration in shallow wells^48–50^.

**Figure 4:**
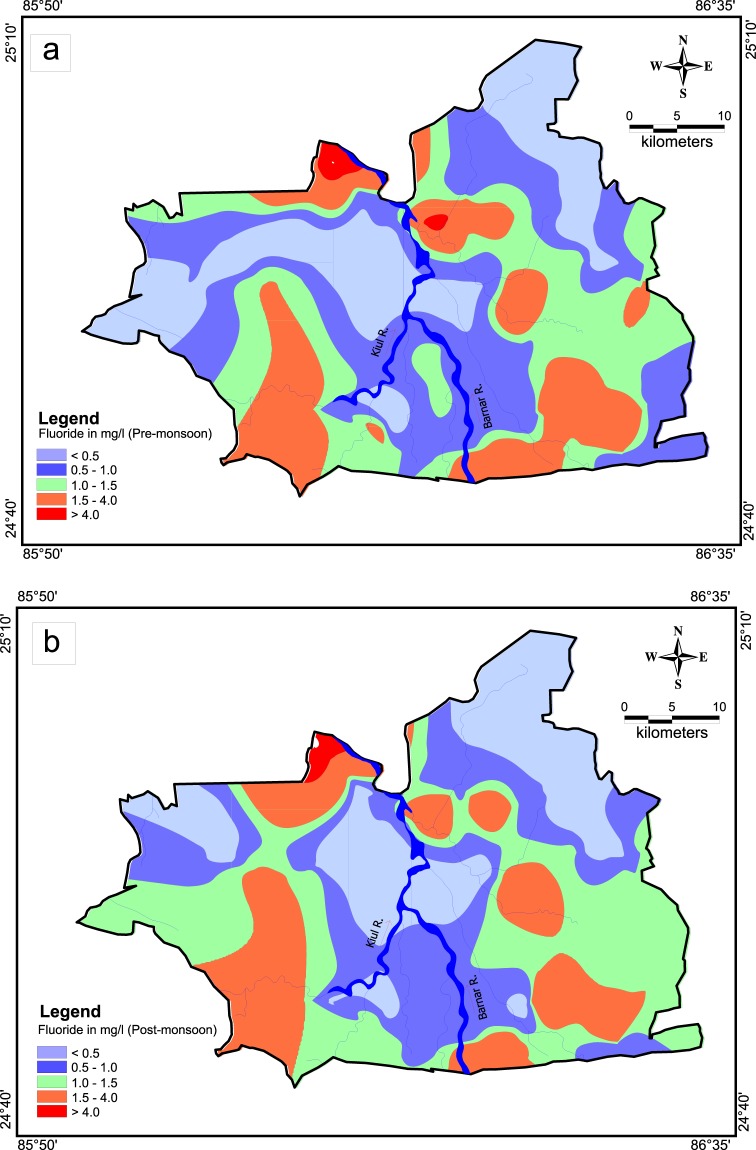
(**a**) Spatial distribution of fluoride in pre-monsoon (**b**) Spatial distribution of fluoride in post-monsoon.

Spatial distribution of fluoride concentration in pre and post monsoon has been shown in Fig. 4a,b, which reveals that concentration of fluoride in the study area is not uniform and this could be plausible due to the control of typical hydrogeological conditions of the study area and water-rock interaction span (i.e., residence time). In general, elevated fluoride concentration is more common in eastern part of the area compared to western part. The fluoride-enriched pockets are mostly associated with alluvium tracks underlain by granite genesis and pediplain of gneissic intrusive rocks. In terms of seasonal variability of fluoride content, pre-monsoon has dominant concentration than post-monsoon and would be the striking observation mainly because of dilution effect due to ample rainfall.

### Petrographic fluoride indicator minerals and governing hydro-chemical mechanisms

**Figure 5:**
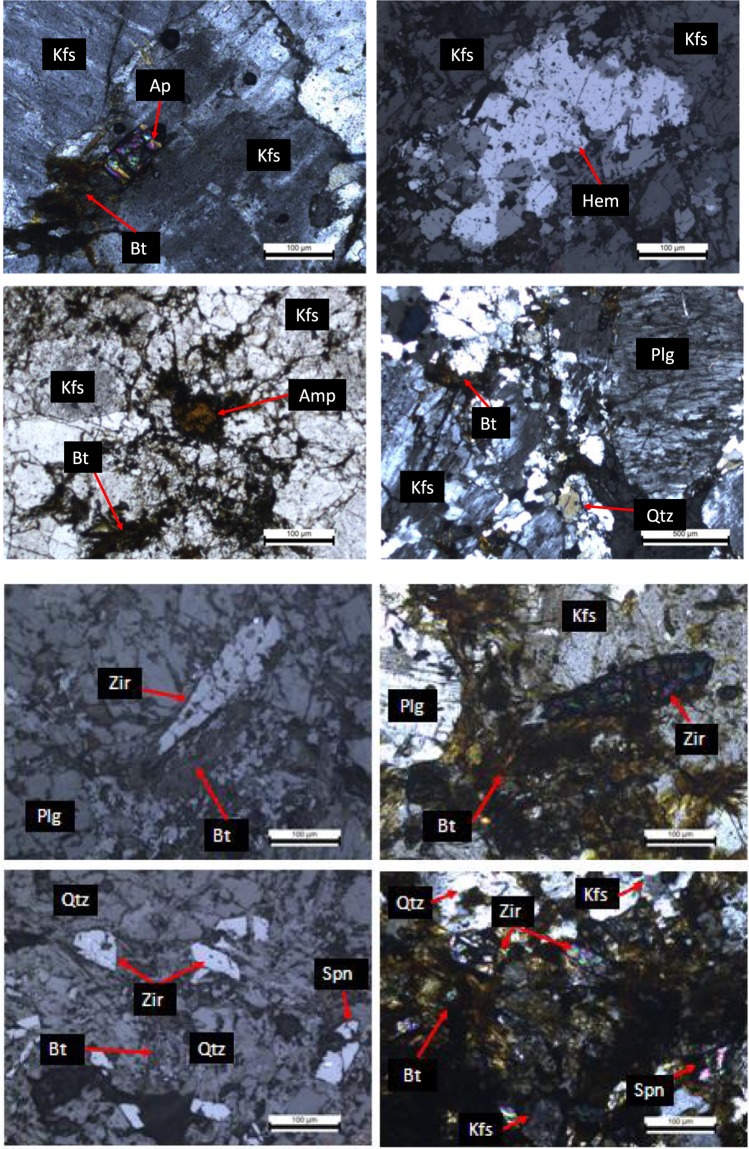
Microscopic observation of rock specimens illustrating different mineral assemblages: Spn-sphene, Ap-apatite, Bt-biotite, Amp-Amphibole, Qtz-quartz, Kfs-potassium feldspar, Zir-zircon.

In the study area, except for the valley-filled materials along the river course and fresh rock exposures over the hill ranges, entire area is occupied by moderately thick weathered mantle, developed due to *in-situ* weathering of crystalline rocks (Fig. 1). Therefore, to establish the possible source of fluoride and its fixation into groundwater, the rock samples were collected from the study area and minerals have been characterized using detailed petrographic studies. Microscopic observation indicates the presence of K-feldspar, plagioclase feldspar, amphibole and biotite in the rock samples collected from Majhwe village (Fig. 5). The fluorine-bearing minerals viz., apatite and sphene have also been observed within the above lithopackage exhibiting different scales of alteration/decomposition. Fe leachates are seen along biotite rich zones and along grain boundaries of feldspar from the samples collected from Panch Pahari area (Fig. 5).

It is clear from the microscopic study that high-fluorine minerals such as amphibole, biotite and apatite (as well as sphene) contribute fluoride to percolating groundwater/ within the aquifer that triggers to high fluoride anomalies in groundwater in areas of high abundance of these minerals. Some of the *in-situ* reactions under alkaline conditions causing dissolution of fluorine bearing minerals namely fluorite, apatite, biotite and hornblende which are responsible for the higher fluoride concentration in the area are as follows^46,51^:

Fluorite (Eq. 7)

7 $${{\rm{CaF}}}_{{\rm{2}}}+2HC{O}_{3}\to CaC{O}_{3}+2{F}^{-}+{{\rm{H}}}_{{\rm{2}}}{\rm{O}}+{{\rm{CO}}}_{{\rm{2}}}$$

Biotite (Eq. 8)

8 $${{\rm{KMg}}}_{{\rm{3}}}[AlS{i}_{3}{O}_{10}]{F}_{2}+2O{H}^{-}\to KMg[AlS{i}_{3}{O}_{10}]\,{[\mathrm{OH}]}_{{\rm{2}}}+2{F}^{-}$$

Hornblende (Eq. 9)

9 $${{\rm{Ca}}}_{{\rm{5}}}\,M{g}_{{\rm{5}}}[S{i}_{6}\,A{l}_{{\rm{2}}}\,{O}_{22}]{F}_{2}+2O{H}^{-}\to {{\rm{Ca}}}_{{\rm{5}}}\,M{g}_{{\rm{5}}}\,[S{i}_{6}A{l}_{{\rm{2}}}{O}_{22}]\,{[\mathrm{OH}]}_{{\rm{2}}}+2{F}^{-}$$

Apatite (Eq. 10)

10 $${{\rm{Ca}}}_{{\rm{10}}}\,{(P{O}_{4})}_{6}{F}_{2}+2O{H}^{-}\to {{\rm{Ca}}}_{10}\,{(P{O}_{4})}_{6}{{\rm{OH}}}_{{\rm{2}}}+2{F}^{-}$$

Muscovite (Eq. 11)

11 $${{\rm{KAl}}}_{{\rm{2}}}[AlS{i}_{3}{O}_{10}]{F}_{2}+2O{H}^{-}\to KA{l}_{2}[AlS{i}_{3}{O}_{10}]{{\rm{F}}}_{{\rm{2}}}+2O{H}^{-}$$

Furthermore, many of the researchers across the world suggested that weathering of fluoride-bearing minerals such as apatite, biotite, mica, sphene, hornblende and various other minerals liberate fluoride into groundwater under favorable conditions (Eqs 7–11)^46,51^. Amongst all, amphibole group is least resistant to weathering and thus probably may add higher amounts of fluoride to groundwater aquifer^52^. Generally, biotite in granite rocks may possess as much as 0.91% fluorine, hornblende (0.71%) and fluorapatite (3.72%)^53^. In a batch dissolution study performed in the laboratory by Chae *et al*.,(2006) established that at room temperature (25 °C), dissolution of biotite enhances fluoride concentration by 100% within 200 hours^54^.

Additionally, humid climate in the study area also enhances the weathering of host rocks and as a result of which, weathered material has developed over granite, granite-gneiss, amphibolite and mica-schist. Fluorine (F^−^) being negatively charged ion and member of the halogen group, is commonly found in such weathered material and will possibly contribute to the groundwater by rock-water interaction mechanism under favorable pH conditions, due to process of selective sorption of OH^−^ to the aquifer matrix, and eventually F^−^ gets released into the solution (Eq. 12).

12 $${\rm{R}}-F+HOH\to R-OH+HF$$

This sort of substitution readily takes place in the aquifer matrix mainly due to the ionic radius of fluoride (133 pm) nearly resembles with that of hydroxyl ion (140 pm)^55,56^. Say for an instance, if the hydrogen ion concentration of less than 7 prevails in the groundwater, F^−^ remains adhered with clay due to the low solubility of F^−^. The reverse is the situation in alkaline medium, where, OH^−^ group replaces the exchangeable F^−^ of clay minerals (biotite/muscovite) resulting in the enhanced F^−^ concentration in aquifer^48,57^.

**Figure 6:**
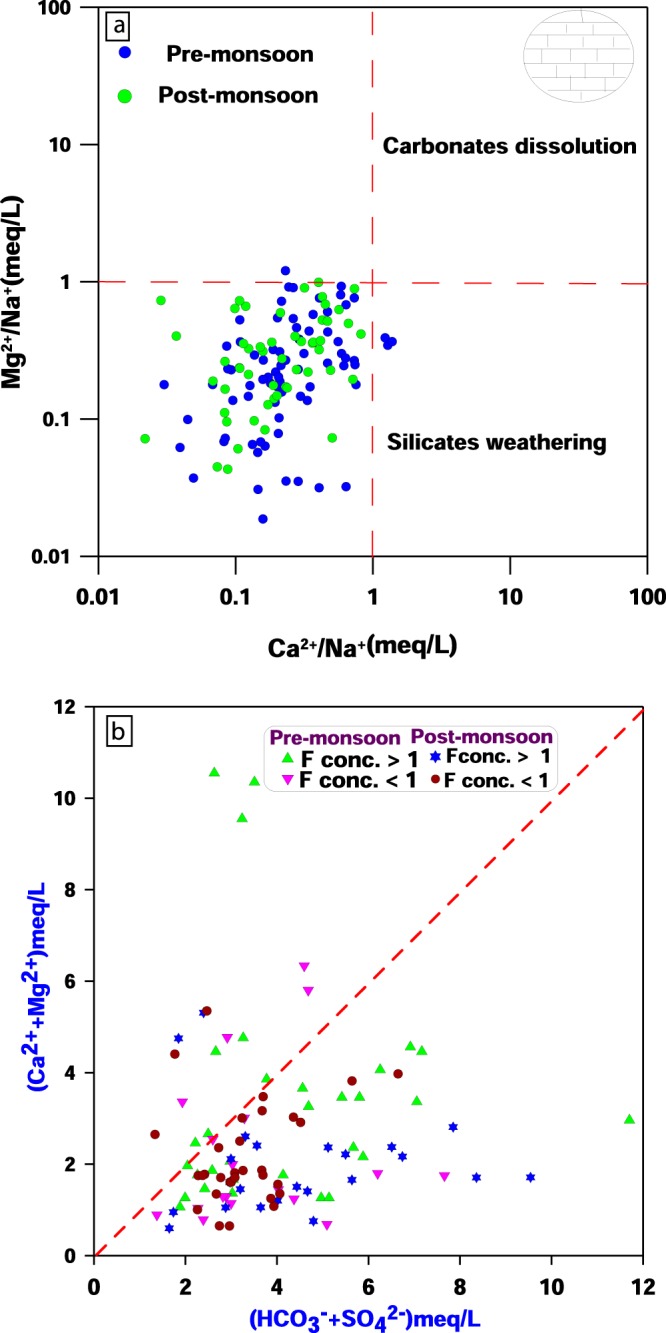
(**a**) Bivariant plot of Mg^2+^/Na^+^ verses Ca^2+^/Na^+^ and (**b**) Cross plot of (Ca^2+^ + Mg^2+^) verses (HCO_3_^−^ + SO_4_^2−^).

Furthermore, bivariant plot of Mg^2+^/Na^+^ verses Ca^2+^/Na^+^(Fig. 6a) clearly exhibits that silicate weathering (granite gneiss) is the dominant process contributing to the chemical quality in the groundwater of the area. Again, observing the cross plot of (Ca^2+^ + Mg^2+^) verses (HCO_3_^−^ + SO_4_^2−^) (Fig. 6b) implies that most of the samples having fluoride concentration more than one in both seasons are placed below equilline, which further confirms silicate weathering and ion exchange in the area is responsible for higher concentrations of F^−^ and HCO_3_^−^ in the solution^31,46,58^. It is a well-established fact that, increase in pH (i.e. alkaline condition), sodium and bicarbonate ion concentration ultimately increases concentration of fluoride in the groundwater due to aforementioned reactions and mechanisms.

In essence, rock-water interaction due to longer residence time and ion exchange process accommodates weathering and leaching of fluoride-bearing minerals in rock formations under alkaline environment thereby adding higher amounts of fluoride to groundwater/ aquifer. This observation is in agreement with the results obtained by others as well^30,31,39^.

### Excess fluoride exposure and health related implications on inhabitants

**Figure 7:**
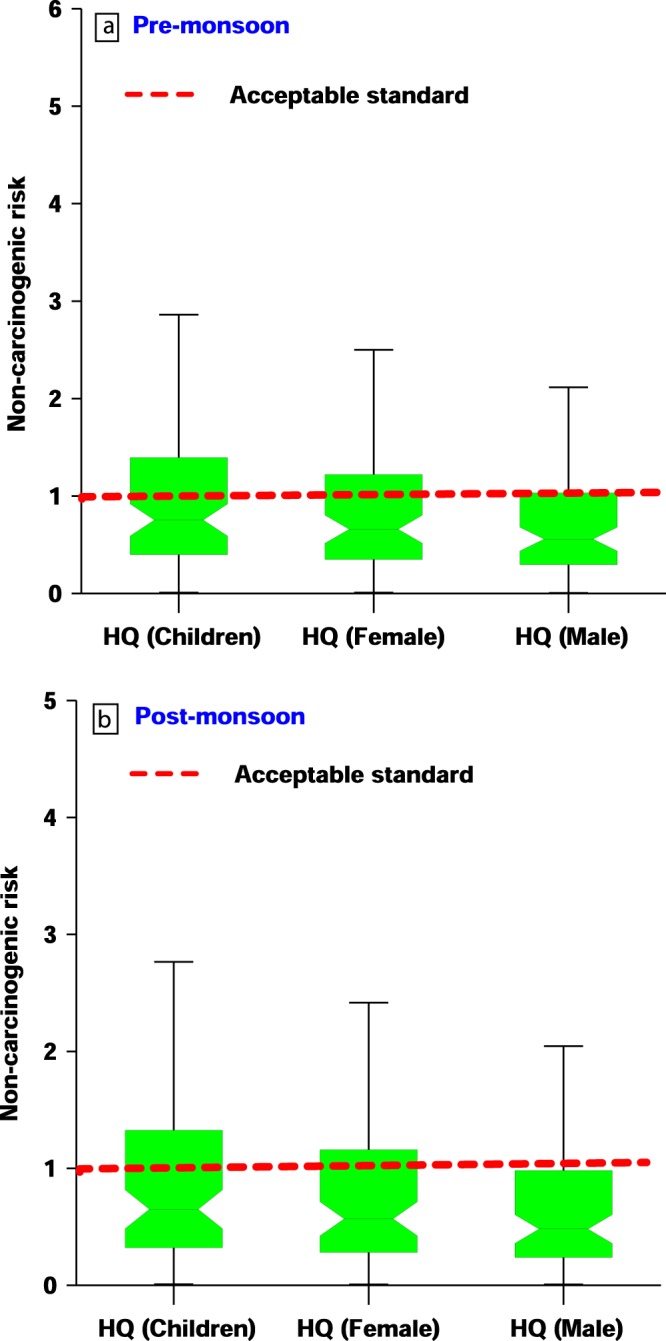
Boxplots showing the result of non-carcinogenic risk for fluoride in (**a**) pre-monsoon and (**b**) post-monsoon.

Health risk assessment is of great importance for estimation of nature and probability of health effect to the inhabitants residing in the areas, which are prone to health hazard contaminants through the drinking water. Amongst all contaminants, oral intake of excess fluoride through drinking water has vital role and may pose non-carcinogenic health risk to population^41^. Accordingly, non-carcinogenic risk of fluoride to human health is estimated in terms of hazard quotient (HQ) and the results of health risk assessment for different age groups is shown in (Fig. 7a,b) and Table 1. Hazard quotient for fluoride in pre and post monsoon season were 0.009–5.02 and 0.009–4.80 with a mean value of 1.13 and 1.01 for children, 0.008–4.39 and 0.008–4.19 with a mean value of 0.98 and 0.88 for female and 0.006–3.71 and 0.006–3.55 with a mean value of 0.83 and 0.75 for males respectively. Children consumers in the study area are more prone to non-carcinogenic risk of fluoride exposure as 32% shallow well and 44% deeper well exhibit HQ value for fluoride more than one in water samples collected before precipitation. During post-monsoon, also, 27% shallow wells and 40% deeper wells have HQ value of fluoride more than one for children. As shown in Fig. 7(a,b), the HQ values for fluoride are in the order of children > females > males for both the seasons. It has been established in various researches that children age group is more prone to non-carcinogenic risk of fluoride as compared to adults^59–61^. Lower body weight of children than adults may be the reason behind higher risk for children^62^. Fluoride exposure in early stages of life is very important as it may prevent caries but may develop dental and skeletal fluorosis. Fluoride intake during first three years of life is most critical in fluorosis etiology^63^.

**Table 1:** Non-carcinogenic risk assessment for children, adult females, and males, in the study region due to excessive fluoride ingestion.

Humans	Type of well	Hazard quotient (HQ)	Health risk	Number of samples	Percentage of samples (Pre-monsoon)	Percentage of samples (post-monsoon)
Males	Shallow	<1	No risk	22	73%	82%
		>1	High risk	22	27%	18%
	Deep	<1	No risk	68	67%	74%
		>1	High risk	68	33%	26%
Females	Shallow	<1	No risk	22	73%	77%
		>1	High risk	22	27%	23%
	Deep	<1	No risk	68	62%	64%
		>1	High risk	68	38%	36%
Children	Shallow	<1	No risk	22	68%	73%
		>1	High risk	22	32%	27%
	Deep	<1	No risk	68	56%	60%
		>1	High risk	68	44%	40%

**Figure 8:**
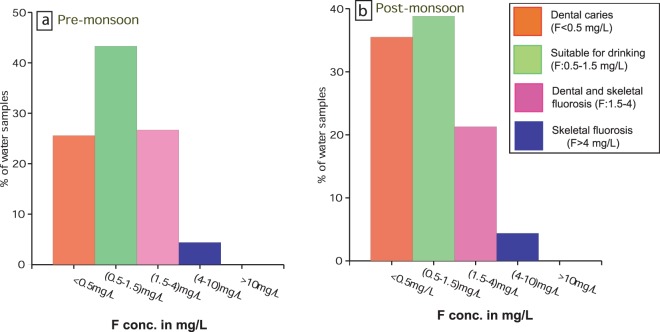
Health risk effects associated with F^−^ ingestion in individuals during different seasons in the study area.

Consumption of drinking water with fluoride concentration more than the prescribed limit of 1.5 mg/L in both seasons may develop dental fluorosis in children and adults. On the basis of fluoride concentration (mg/L), groundwater of the study area has been divided into five classes. A total of 43% and 38% groundwater samples fall in Class-II and can be considered safe for drinking purpose. Whereas, 30% sample in pre-monsoon and 25% groundwater samples in post-monsoon falls under class-III and class-IV and may develop dental and skeletal fluorosis in consumers^64^ (Fig. 8a,b).

**Figure 9:**
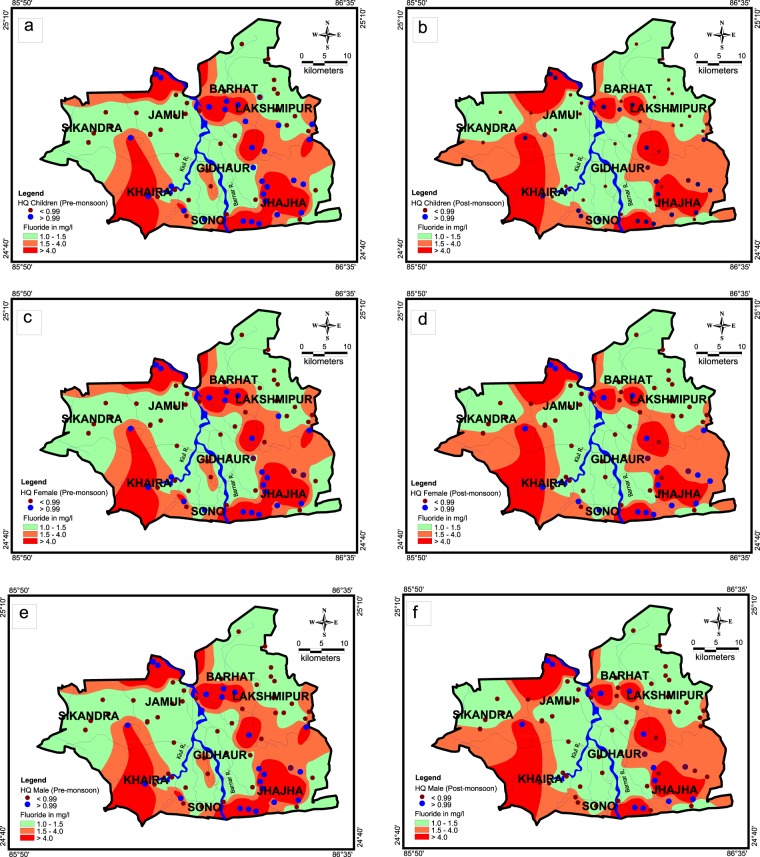
Spatial-temporal distribution of HQ along with F^−^ during pre-monsoon (**a**) HQ Children, (**b**) HQ Female, (**c**) HQ Male and post-monsoon season (**d**) HQ Children, (**e**) HQ Female, (**f**) HQ Male.

The spatial distribution of HQ for different age groups in the pre and post monsoon has been shown in Fig. 9(a–f). Scenario is worst in villages of Jhajha block, which is situated in south eastern part of the study area. In Jhajha block, 63% and 68% samples exhibit HQ value of fluoride for children consumers more than unitary in pre and post monsoon respectively. HQ value of fluoride for females is more than one in 58% and 64% of water samples in pre and post monsoon respectively. Fluoride concentration of more than 1.5 mg/L has been found in deeper as well as shallow aquifer in the groundwater of Jhajha block (Fig. 3) mainly because of geology (i.e. most of the part is covered by granite, gneisse and pegmatite veins) and geomorphology of the block that eventually leads to elevated HQ values in this block. In Jakotia, Loha, New Abadiganj, Lakraha, Gopalpur, Harna and Bhadwaria villages of Jhajha block, skeletal and dental fluorosis has been noticed on large scale in children and adults. Similarly, in Jamui block most of the area comprises of alluvium formation, except Majhwe village which is situated in extreme north and is surrounded by granite hillocks from three sides. In Majhwe village, HQ value for fluoride is 2.86 to 4.80. In the groundwater samples collected from the paired wells of this village, fluoride concentration was found 4.80 mg/L and 3.60 mg/L for shallow and deeper aquifers respectively (Fig. 3). A field survey conducted in fluoride affected villages of Jhajha and Jamui blocks reveal that habitants exposed to mild dosage of fluoride contaminated water for lesser time cause discoloration of teeth with white or yellow patches in the area, whereas, consumption of excessive fluoride content water for the prolonged duration triggers to bone deformities or else known as skeletal fluorosis.

Northern part of the Laxhmipur block which is situated in north eastern part of the study area is covered with quartzite, phyllites and alluvium formation, has been found with lesser concentration of fluoride in groundwater. On the other hand, southern part of the Laxhmipur block in the proximity of granite gneiss terrain of Jhajha block is the area of elevated fluoride concentration vis-a-vis higher HQ values (>1). In the alluvium formation of Khaira and Sikandra block, fluoride concentration is high neither in shallow nor in deep aquifer, except in the well near granite gneiss in the proximity of Gidheswar hills.

### Non-carcinogenic vs. carcinogenic fluoride issue debate

In general, minerals play essential roles in the normal metabolism and physiological functions of animals and humans. Amongst, fluorine, occurring in groundwater as the fluoride (F^-^) ion is essential in small quantities for structural functions in bone and membranes (0.5–1.0 mg/L)^31,65^. Despite its usefulness, chronic ingestion of excessive F^−^ water can cause fluorosis of the enamel and bone (i.e. 1.5–4 mg/L) and in extreme cases, skeletal fluorosis associated with joint stiffness, calcification of ligaments, and some osteosclerosis of the pelvis and vertebrae (i.e. 4–10 mg/L)^3,4,66^. This happens mainly because of the F^−^ being highly electronegative and having similar ionic radius (133 pm) with that of hydroxyl ion (140 pm) that leads to the formation of hydrogen fluoride^48,55–57^. Particularly, this compound in human body freely diffuses across the intestine, dissolves in the blood, and accumulation in calcified tissues^7,21^. Therefore, health related issues in and around fluoride affected areas are mostly non-carcinogenic in nature, especially from the studied areas, when fluoride in drinking water ranges not more than 10 mg/L^67,68^. However, higher doses (>10 mg/L) have also been linked to crippling fluorosis and carcinogenic potential^66–69^ although it is not established completely^12,70,71^. Kharab *et al*.(2012)^24^ in their study suggested some possible link with fluoride in drinking water to osteosarcoma. The limited studies on this aspect suggest that fluoride could cause cells to grow faster so that they may become cancerous in course of time, albeit debatable as no credible link exists to suggest the linkage between fluoride and effect of carcinogenicity^23,25^. As defined by the National Institute of Cancer (NIC) of USA, the osteosarcoma is linked to the bone cancer affecting large bones of arms and legs and it is prevalent in younger generation especially more in males to females (https://www.cancer.gov/publications/dictionaries/cancer-terms/def/osteosarcoma)^72^. However, the theory is yet to be established and is still of considerable debate today. Whereas, non-carcinogenic related health effects are very much prevalent across the world and in particular in the studied areas, starts from dental to skeletal fluorosis related problems affecting more children followed by adult females as compared to adult males^73–75^.

### Concluding remarks and possible remedial thoughts

In the study area, most of the groundwater samples from hard rock formations possess fluoride concentration more than the permissible limit of 1.5 mg/L. On the other hand, groundwater samples from alluvial formations have less incidence of fluoride concentration of more than the permissible limit. Microscopic analysis of rock samples in the study area confirms the presence of fluoride-bearing minerals, for instance, biotite, apatite, quartz, sphene and hornblende. Weathering of these fluoride bearing minerals developed over granite, granite-gneiss, amphibolite and mica-schist is the prominent factor contributing to higher fluoride concentration in the study area. Mg^2+^/Na^+^ versus Ca^2+^/Na^+^ and (Ca^2+^ + Mg^2+^) verses (HCO_3_^−^ + SO_4_^2−^) plot further confirms that silicate weathering and ion-exchange are the dominant processes involved for the higher concentration of fluoride and bicarbonate ions in the area. The non-carcinogenic health risk assessment (HQ) reveals that children are more prone to the risk of fluoride exposure followed by females and males.

The investigation of fluoride concentration indicates that about 30% groundwater samples in pre-monsoon and 25% groundwater samples in post-monsoon fall under class-III and class-IV and, as a consequence, may develop dental and skeletal fluorosis in consumers. Symptoms of dental and skeletal fluorosis have been reported in majority of habitantsfrom the entire Jhajha block, northern part of the Lakshmipur block and Sono blocks situated in the south eastern part of the study area, where HQ exceeds the unitary value alongside F concentration >1.5 mg/L. Symptoms of dental and skeletal fluorosis have also been found in the population of Majhwe and Navinagar villages of Jamui block, situated in the north-western parts of the study area. Except Majhwe and Navinagar villages which are surrounded by granite hills from three sides, the remaining areas of Jamui block are the part of alluvium formation. However, in the groundwater resources of Krishi Kendra, Khadigram of Barhat block, fluoride concentration was within the permissible limit of 1.5 mg/L, although this area is surrounded by fluoride-affected habitations. Five ponds situated in the premises of Krishi Vigyan Kendra might have been helpful in diluting the enhanced fluoride concentration. Therefore, revival of ponds and rainwater harvesting structures in the affected area may improve the quality of potable water by diluting the fluoride concentrations in the groundwater resources. Fortunately, there are large surface water reservoirs like Nakti Dam, Nagi Dam and, Belatand Dams in the study area which have a fluoride concentration within the permissible limit of 1.5 mg/L. Drinking water may be supplied from these surface water reservoirs to affected habitations so that adverse health effects can be prevented. Further, it is also recommended that policymakers ought to adopt solar energy-based electrolytic de-fluoridation technology in these fluoride affected villages and regular monitoring of supplying drinking water quality apart from that of habitants health condition/implications.

## Supplementary information

Supplementary Information
