# Supplementary material for: Medical Geological assessment of fluoride contaminated groundwater in parts of Indo-Gangetic Alluvial plains
Source: Sci Rep. 2019 Nov 7;9:16243. doi: 10.1038/s41598-019-52812-3 (PMC6838337; doi:10.1038/s41598-019-52812-3)

**Supplementary Information for “Medical Geological assessment of fluoride contaminated groundwater in parts of Indo-Gangetic Alluvial plains”**

Suresh Kumar^1&3^, Rambabu Singh^2^, A. S. Venkatesh^3^*, G. Udayabhanu^4^, P R Sahoo^3^

^1^Central Ground Water Board, Patna, India

^2^Central Mine Planning and Design Institute Limited, Bilaspur, India

^3^Department of Applied Geology, Indian Institute of Technology

(Indian School of Mines), Dhanbad, India

^4^Department of Applied Chemistry, Indian Institute of Technology

(Indian School of Mines), Dhanbad, India

*Corresponding author: asvenkatesh@iitism.ac.in

**List of supplementary tables**

Table S1: Parameters used for probable fluoride risk assessment

| **Variable** | **Abbreviation** | **Unit** | **References** | **Men** | **Women** | **Children** |
| --- | --- | --- | --- | --- | --- | --- |
| Groundwater fluoride concentration | CW | mg/L |  |  |  |  |
| Drinking water ingestion rate | IRW | L/day | USEPA, 2014^65^ | 2.5 | 2.5 | 0.78 |
| Exposure frequency | EF | days/year | USEPA, 2014^65^ | 365 | 365 | 365 |
| Exposure period | ED | years | Adimalla and Li, 2018^66^ | 64 | 67 | 12 |
| Average body weight | BW | kg | ICMR, 2009^67^ | 65 | 55 | 15 |
| Average exposure time | AT | days | WHO, 2013^68^ | 23,360 | 24,455 | 4380 |
| Oral reference dose | RfD | mg/kg/day | Fallahzadeh et al., 2018^6^ | 0.06 | 0.06 | 0.06 |

Table S2: Statistical summary of season and depth wise chemical composition of groundwater and related health effects.

| **Chemical Constituent** | **Type of well** | **Pre-monsoon** | | | **Post –monsoon** | | | **Desirable limit** | **Permissible Limit** | **Potential health effect** |
| --- | --- | --- | --- | --- | --- | --- | --- | --- | --- | --- |
|  |  | **Minimum** | **Maximum** | **% of the sample below permissible limits** | **Minimum** | **Maximum** | **% of the sample below permissible limits** |  |  |  |
| pH (Units) | Shallow | 6.8 | 8.66 | 89% | 6.66 | 8.6 | 73% | 6.5-8.5 | No relaxation | Taste, corrosion |
|  | Deep | 6.28 | 8.52 | 94% | 7.08 | 8.6 | 92% |  |  |  |
| EC(µS/cm) | Shallow | 325 | 2317 | 83% | 572 | 2210 | 60% | _ | 1500^*^ | Gastrointestinal irritation |
|  | Deep | 323 | 2344 | 91% | 157 | 1495 | 89% |  |  |  |
| TDS(mg/L) | Shallow | 211 | 1506 | 100% | 371 | 1436 | 100% | 500 | 2000 | Gastrointestinal irritation |
|  | Deep | 210 | 1524 | 100% | 157 | 1495 | 100% |  |  |  |
| Na (mg/L) | Shallow | 31.33 | 261 | 56% | 36 | 332 | 87% | _ | 200^*^ | Hypertensive effects |
|  | Deep | 19.88 | 401.32 | 87% | 28 | 314 | 83% |  |  |  |
| K (mg/L) | Shallow | 0.13 | 139.92 | 100% | 0.78 | 81.65 | 73% | _ | 12^*^ | Bitter taste |
|  | Deep | 0.1 | 41.59 | 89% | 0.74 | 73.17 | 89% |  |  |  |
| Ca (mg/L) | Shallow | 10 | 212 | 100% | 10 | 182 | 100% | 75 | 200 | Scale formation |
|  | Deep | 4 | 68 | 100% | 12 | 166 | 100% |  |  |  |
| Mg (mg/L) | Shallow | 6.07 | 78.97 | 100% | 3.64 | 57.1 | 100% | 30 | 100 | _ |
|  | Deep | 2.43 | 24.77 | 100% | 1.2 | 49.81 | 100% |  |  |  |
| SO_4_ (mg/L) | Shallow | 0.1 | 162 | 100% | 12.7 | 122.76 | 100% | 200 | 400 | Laxative effect |
|  | Deep | 0.1 | 165.79 | 100% | 2.9 | 136.76 | 100% |  |  |  |
| HCO_3_ (mg/L) | Shallow | 18.45 | 393.6 | _ | 55.35 | 559.65 | _ | _ | _ | _ |
|  | Deep | 12.3 | 694.95 | _ | 69 | 485.85 | _ |  |  |  |
| TH(mg/L) | Shallow | 50 | 630 | 100% | 65 | 535 | 100% | 200 | 600 | _ |
|  | Deep | 55 | 530 | 100% | 60 | 530 | 100% |  |  |  |
| NO_3_ (mg/L) | Shallow | 1 | 50.77 | 83% | 0.1 | 158 | 40% | 45 | No relaxation | Methemoglobinemia |
|  | Deep | 0.1 | 56 | 94% | 0.1 | 180 | 58% |  |  |  |
| Cl (mg/L) | Shallow | 10.63 | 553.02 | 100% | 10.3 | 555 | 100% | 250 | 1000 | Anaesthetic effect, Salty taste |
|  | Deep | 7.09 | 567 | 100% | 12.3 | 596 | 100% |  |  |  |
| F(mg/L) | Shallow | 0.08 | 4.8 | 81% | 0.02 | 5.54 | 82% | 1 | 1.5 | Skeletal and dental fluorosis |
|  | Deep | 0.01 | 5.8 | 66% | 0.01 | 4.65 | 72% |  |  |  |

Note: * implies WHO, 2004^27^ standards.

**Supplementary figures list**

Figure S1: Comparison of fluoride distribution in groundwater of the study area for the year 2014 vs 2018.

**
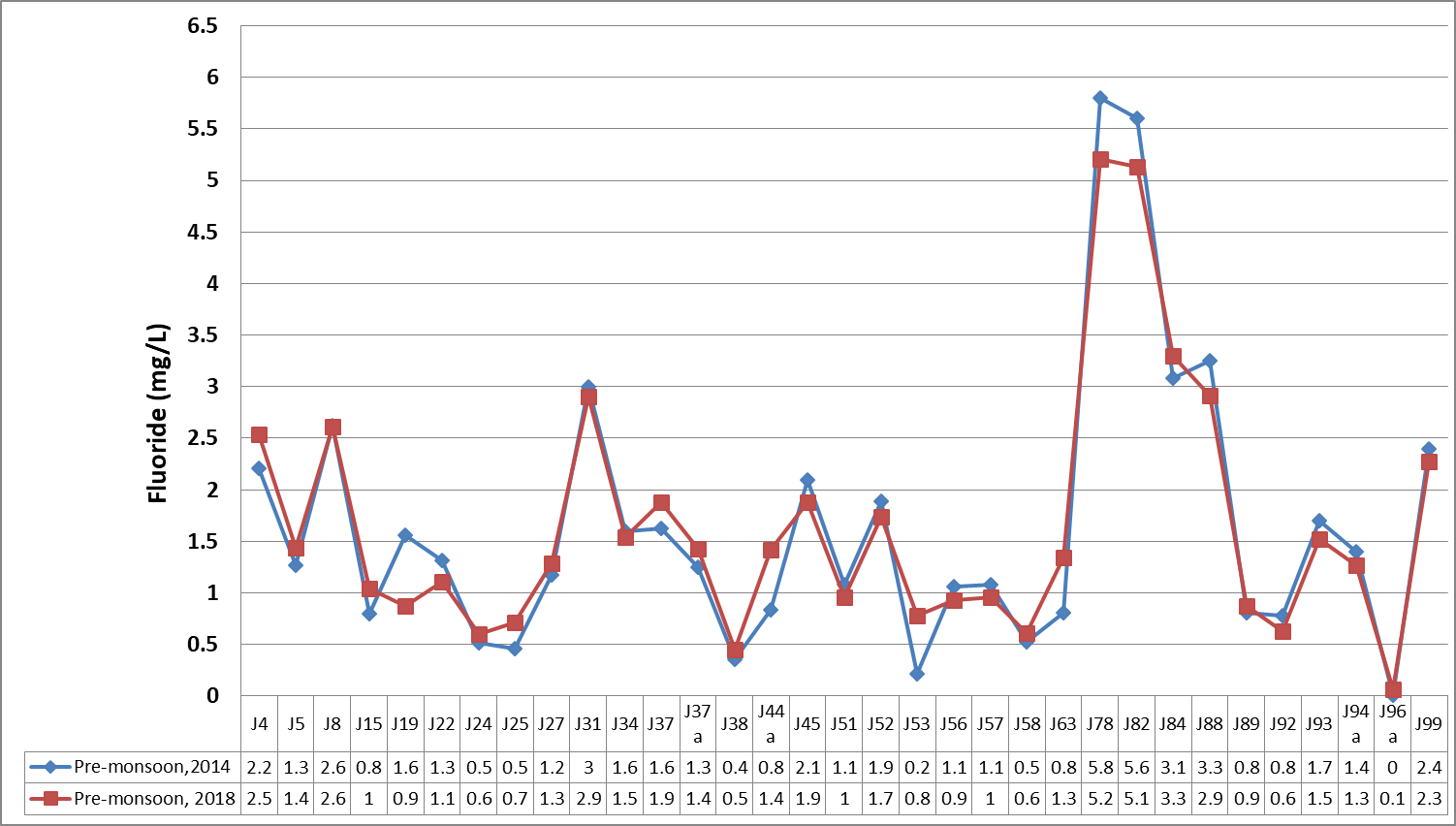
**

Figure S2: Fluoride distribution in (a) shallow aquifer and (b) deeper aquifer for pre-monsoon season.


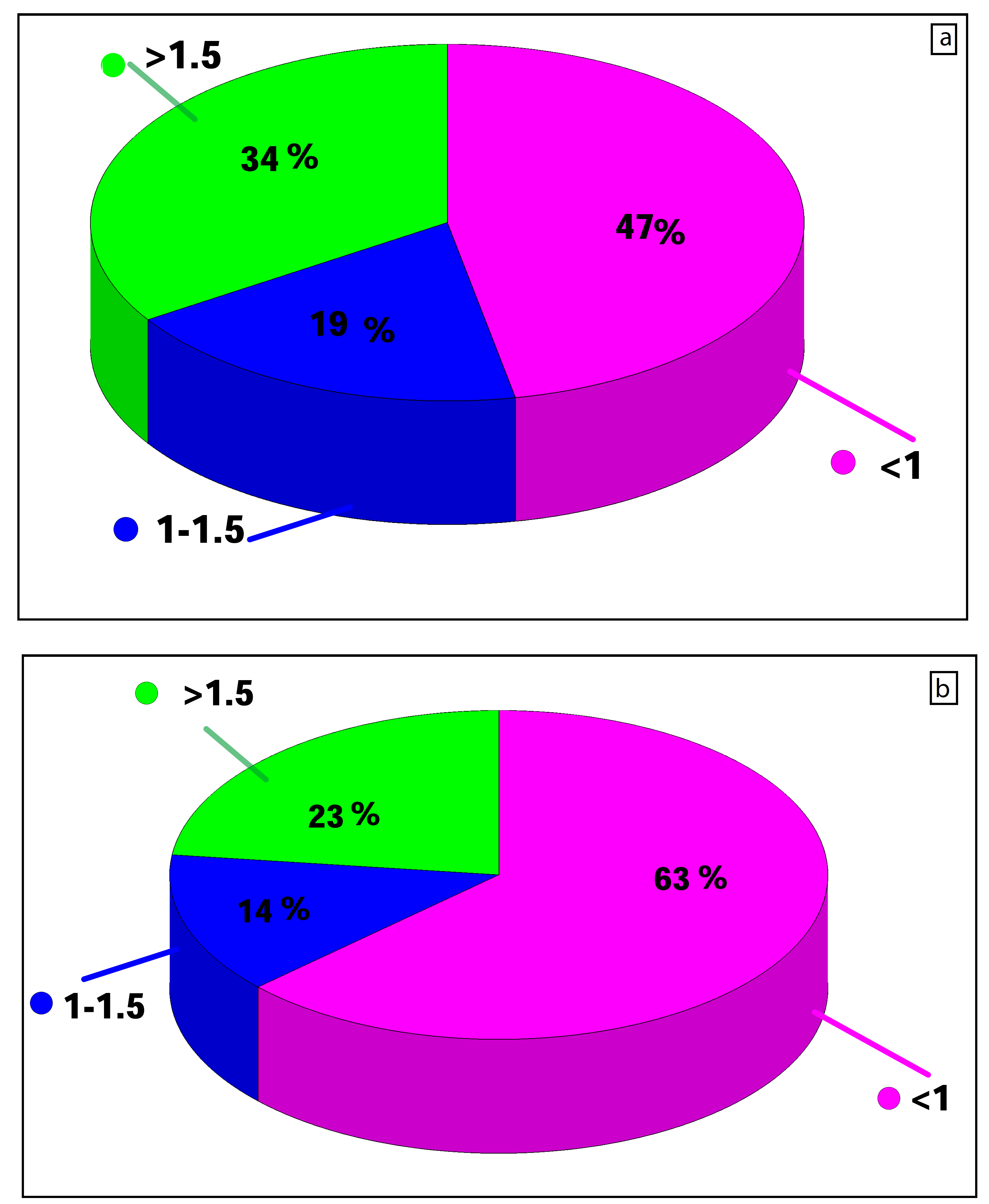

Supplement: Supplementary file 1 — Supplementary Information [file 41598_2019_52812_MOESM1_ESM.doc]
